# Effects of disturbances on aboveground biomass of alpine meadow in the Yellow River Source Zone, Western China

**DOI:** 10.1002/ece3.8640

**Published:** 2022-03-08

**Authors:** Yan Shi, Jay Gao, Xilai Li, Jiexia Li, Gary Brierley

**Affiliations:** ^1^ 1415 School of Environment The University of Auckland Auckland New Zealand; ^2^ 207475 State Key Laboratory of Plateau Ecology and Agriculture Qinghai University Xining China

**Keywords:** AGB change, alpine meadow, disturbance intensity, Qinghai‐Tibet Plateau, sole and joint impacts

## Abstract

A field experiment quantifies the impacts of two external disturbances (mowing‐simulated grazing and number of pika) on aboveground biomass (AGB) in the Yellow River Source Zone from 2018 to 2020. AGB was estimated from drone images for 27 plots subject to three levels of each disturbance (none, moderate, and severe). The three mowing severities bear a close relationship with AGB and its annual change. The effects of pika disturbance on AGB change were overwhelmed by the significantly different AGB at different mowing severities (−.471 < *r* < −.368), but can still be identified by inspecting each mowing intensity (−.884 < *r* < −.626). The impact of severe mowing on AGB loss was more profound than that of severe pika disturbance in heavily disturbed plots, and the joint effects of both severe disturbances had the most impacts on AGB loss. However, pika disturbance made little difference to AGB change in the moderate and non‐mowed plots. Mowing intensity weakens the relationship between pika population and AGB change, but pika disturbance hardly affects the relationship between mowing severity and AGB change. The effects of both disturbances on AGB were further complexified by the change in monthly mean temperature. Results indicate that reducing mowing intensity is more effective than controlling pika population in efforts to achieve sustainable grazing of heavily disturbed grassland.

## Graphical abstract

This study quantified the effects of disturbances on AGB and evaluates the importance of pika and mowing impacting on AGB change individually and jointly for the first time. These results will help us assess the disturbance pressure and develop sustainable grazing strategies to protect this vulnerable ecosystem.

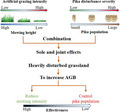

## 1 INTRODUCTION

Yak and pika are key determinants of the functionality of grazing adapted grassland ecosystems, playing an irreplaceable role in maintaining the integrity of the vulnerable ecosystems on the Qinghai‐Tibet Plateau (QTP) (Long et al., 2008). Alpine grassland occupies an area of around 2.5 million km^2^ on the QTP (Li, Gao, et al., 2013), providing an important grazing resource that supports the livelihood of tens of thousands of pastoralists (Chen & Zhang, 2000). In recent decades, overgrazing and rodent outbreaks have decreased grassland aboveground biomass (AGB) (Dong & Sherman, 2015), which not only governs the quantity of forage production but also reflects livestock carrying capacity (Tao et al., 2005; Zhu et al., 2005). These factors occur alongside climate changes, collectively influencing the condition of grazing adapted ecosystems and impacting upon the sustainability of prevailing land‐use practices in this region (Harris, 2010).

Grasslands on the QTP have been grazed for millennia (Li, 1997). Although grazing can improve grassland health by promoting the diversity of plant species (Wang et al., 2005; Wilcox et al., 2017) and stimulating grass growth by boosting the root system (Yan et al., 2020), it adversely influences grassland vegetation after plant biomass is removed and consumed by livestock (Biondini et al., 1998; Hik & Jefferies, 1990). In recent decades, grasslands in the Yellow River source zone of the QTP have been overgrazed, at an estimated ratio of 67.88% in 2010, or an equivalent of 6.52 million sheep units overloaded (Zhang et al., 2014). Overgrazing is regarded as the main cause of grassland degradation, reducing plant biodiversity and productivity (Dong et al., 2020; Török et al., 2018). Conversely, grazing exclusion is effective at restoring degraded alpine grassland (Liu et al., 2020; Wu et al., 2019). Determining and implementing an appropriate grazing intensity is a key management strategy to support the sustainable use of grasslands (Deak et al., 2017; Li et al., 2017). Grazing intensity refers to the cumulative effects of livestock on grassland, mainly represented by forage intake, over a given time period in a given area (Holechek et al., 1998). It directly affects vegetation structure, coverage, and biomass volume (Toth et al., 2018).

Although plateau pikas (*Ochotona curzoniae*) may not devour as much grassland biomass as livestock, they do more damage to grassland roots and play a more important role in enlarging bare patches within the grassland (Chen et al., 2017). If controlled under an appropriate population, pikas are ecosystem engineers, exerting beneficial effects on plant richness and soil mixing (Pang & Guo, 2018; Yu et al., 2017; Zhou et al., 2018). In fact, they have been considered as a keystone animal of grassland ecosystems (Hogan, 2010). However, at an average density of 429 individuals/km^2^ on the QTP, they consume about 136 million tons of fresh grass annually, enough to feed 150 million sheep (Sun et al., 2016). Apart from direct consumption of fresh forage, pikas also affect AGB via burrowing and gnawing, both of which can damage grassland by piling the loosened soil atop healthy grasses and creating bare patches, thus accelerating grassland degradation which is hard for plant regrowth and recovery. (Li et al., 2021; Li, Perry, et al., 2013; Qin et al., 2020; Wu et al., 2015).

All ecosystems are impacted by global climate change (McMichael et al., 2004), but alpine grasslands on the QTP are especially sensitive to changing temperature and precipitation (Ma et al., 2010; Zha et al., 2005; Zhong et al., 2019). In this region, warmer temperatures can prolong the growing season by advancing the start date and delaying the end date of growth, boosting AGB production (Sun, 2020; Zhang et al., 2013; ; Zhang, Kong, et al., 2018). However, the dry climate is a major limiting control upon grass growth (Dai et al., 2019; Gao et al., 2016). From 2004 to 2016, an increase in air temperature by 0.44°C while precipitation remained stable in northern Qinghai Province was accompanied by a significant increase in belowground biomass of the alpine grassland, but AGB hardly changed (Dai et al., 2019). In general terms, grazing decreases AGB but climate warming increases AGB (Zhang et al., 2015), both of which are further compounded by pika disturbance. Thus, the achievement of long‐term sustainable grazing requires consideration of multiple stressors, assessing impacts of both livestock grazing and pika disturbance, and their interactions, in light of climate change (Brotherton & Joyce, 2015; Li et al., 2019, 2020; Pech et al., 2007).

To date, researchers have studied the effects of different levels of plateau pika disturbance on plant biomass (Pang & Guo, 2018) and the effect of grazing on grassland biomass productivity (Zhang, Wang, et al., 2018). However, there are limited understandings of how the inter‐annual fluctuations in AGB are related to different levels of grazing and pika disturbances. This study quantifies the singular and combined effects of artificial grazing and pika disturbance severities on AGB and its changes in an alpine grassland on the QTP, assessing the relative importance of both disturbances. The specific objectives are to (a) quantify the effects of three pika disturbance severities (controlled by population) and three mowing‐simulated grazing disturbance severities on AGB and its change, (b) evaluate the importance of pika and mowing disturbances on AGB change individually and jointly, and (c) to contextualize the influence of the disturbances on AGB against the backdrop of climate change.

## 2 MATERIAL AND METHODS

### 2.1 Study site

**FIGURE 1:**
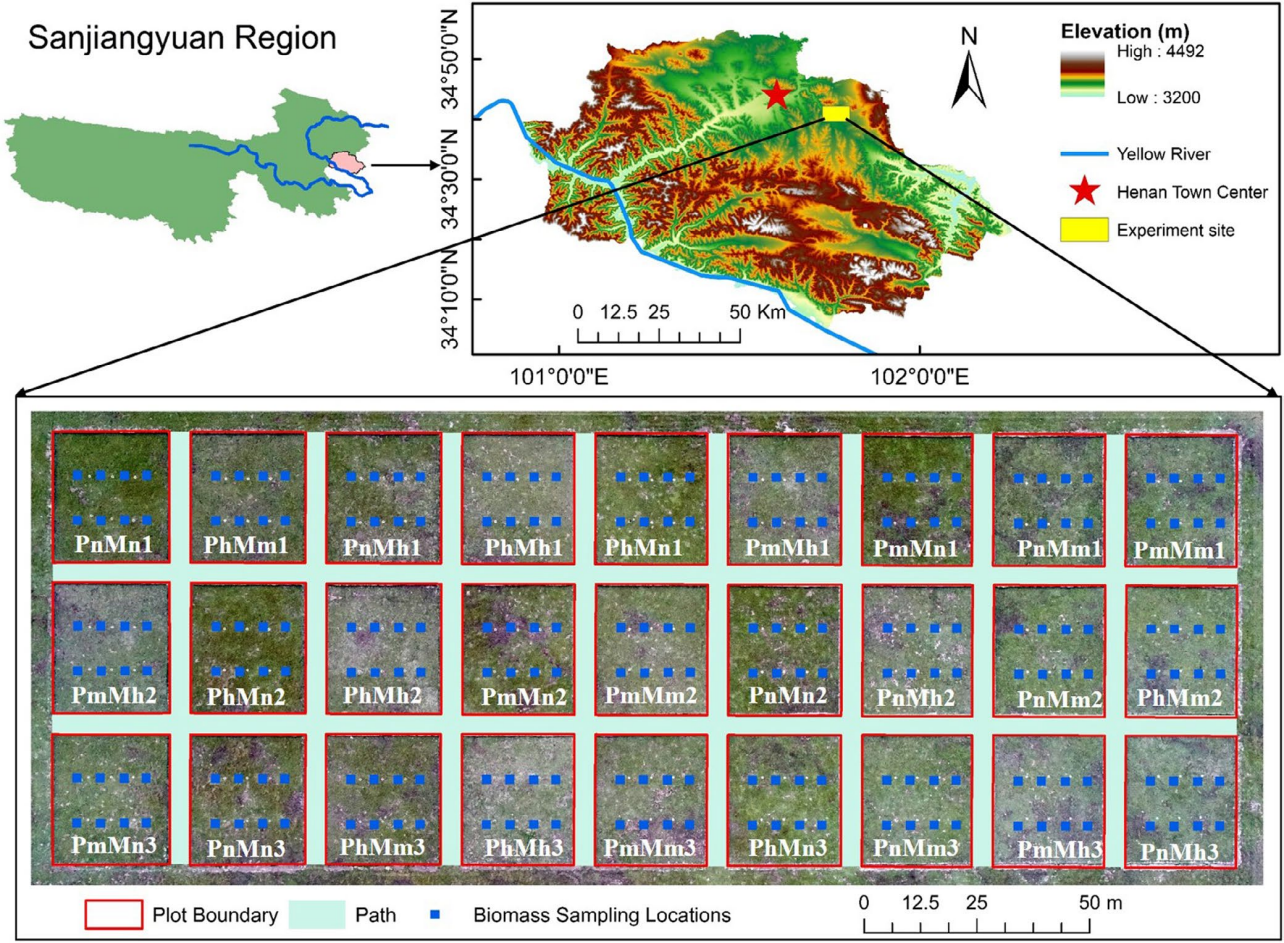
Experiment site location and treatments (1, 2, 3 are replications)

The study site was located on a flat floodplain of the Yellow River near the Henan County town center on the QTP (101°47′E, 34°44′N) (Figure 1). It has an altitude of 3743 m above sea level with a plateau continental climate. Annual temperature ranges from −1.3 to 1.6°C, and annual precipitation ranges from 597.1 to 615.5 mm (http://data.cma.cn/). This area has been affected by pikas and overgrazed for decades (Du et al., 2019; Zhang, Fan, et al., 2018).

### 2.2 Experimental setup

**FIGURE 2:**
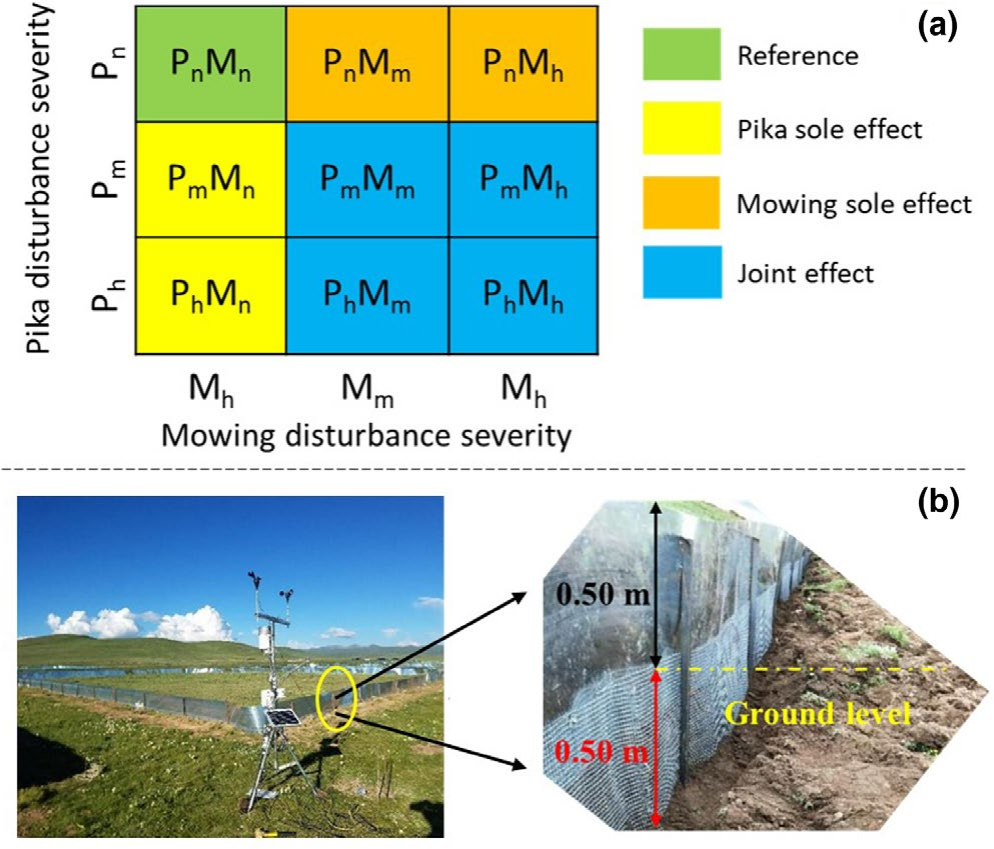
Field experiment design (a is the design of treatments, b is the installation of fence and weather station)

Livestock grazing was simulated via mowing in this study (Tälle et al., 2015). This enables careful and specific regulation by setting the mower blade to different heights (Jerrentrup et al., 2014). Pika disturbance severity was made proportional to its population. Both pika (*P*) and mowing (*M*) disturbances were set at three levels (none (n), medium (m), and high (h)). Of the nine treatments, one served as the reference, two were subject to the sole effect of pika and mowing, and the remaining four plots were subject to joint influences of both disturbances (Figure 2a). Each treatment was replicated three times randomly in three rows, resulting in a total of 27 plots, 25 m × 30 m in size. All plots were spatially disconnected with a 5 m corridor between them. Each plot was fenced with a 50 cm high iron sheet above the ground to prevent pikas from roaming among the plots (Figure 2). A mesh fence of 50 cm was installed underground (mesh diameter of 2.5 cm × 2.5 cm), deeper than pika's burrowing depth of 20–30 cm to prevent pika intrusion and loss (Wei et al., 2007; Xiao et al., 1981).

Plateau pikas are widely scattered in the alpine grassland of the QTP (Hoffmann et al., 2013). Small mammals used in this study weighted 130–200 g. Based on previous research in an alpine meadow near this study (Han et al., 2008; Sun, 2008), the highest pika density (*P*_h_) was set at about 200 individuals/ha, equivalent to 14 individuals per plot. The medium severity (*P*_m_) was set at seven individuals per plot, and no pika plots served as the reference (*P*_n_). Before setting the pika population, traps were deployed in each plot for one month to capture and remove existing pikas, and the reference plots were further treated with poisons to eradicate any residual pikas. All the trapped pikas were later released to the pre‐designated plots at a sex ratio of 1:1 (male vs female) for the *P*_h_ plots and 3:4 for the *P*_m_ plots (Dobson et al., 1998). Pika number and sex ratio were reassessed annually, and any deviation from the experiment design was remedied accordingly. Grazing disturbance was simulated at three levels of high, moderate, and none by machinery mowing at the beginning of July and August of each year. High mowing severity (*M*_h_) was achieved by clipping almost all the AGB by setting the mower blade to the lowest level (about 2 cm above the ground), medium mowing severity (*M*_m_) was achieved by removing half of the plant (about 10 cm above the ground). *M*_n_ plots were not mowed. Residues of the mowed plants were removed from the plots. Blade height was set based on the one‐third principle to reduce stress to the remaining plants, namely, the mowed grass height was less than one‐third of the total grass height at one time (Law et al., 2016). Thus, it would take 2–4 times (2–3 days interval) for the mowed grass to reach the designed height. To model the effect of mowing on AGB quantitatively, mowing severity was quantified at a scale of 0.0 (*M*_n_)‐1.0 (*M*_h_), and a value of 0.5 was assigned to *M*_m_.

### 2.3 Field data collection and processing

The 27 experimental plots were photographed at an altitude of around 40 m using an RGB digital camera (1 inch, 20‐megapixel Complementary Metal Oxide Semi‐conductor sensor) aboard a Dajiang Phantom 4 UAV (Shenzhen Dajiang Baiwang Technology Co., Ltd., Shenzhen, China) in the auto mode in late August each year. The images were not radiometrically corrected, even though this could enhance the accuracy of VI‐derived AGB (De Carvalho et al., 2013). This omission is permissible because VI can be reliably estimated from non‐calibrated high‐resolution images so long as they are obtained under the same radiometric conditions (Bolten et al., 2015). After eight 1 m × 1 m quadrats in each plot were selected (Figure 1), plant biomass in a random quarter of the quadrat was harvested by manually clipping all the plants as close to the ground as possible. The fresh biomass was oven‐dried at 80 ℃ for 48 h and weighted in the lab (dry matter content is 40.31% in this study). The dry weight was regarded as the ground truth biomass for AGB modeling. In total, 216 biomass samples (8 quadrats by 27 plots) were collected each year. A micro weather station (Figure 2b) was set up at the experiment site to collect climate data.

The plot fence, which was clearly visible in the UAV images, served as the ground control for their geo‐referencing to a rectangular size of 25 m by 30 m in ArcMap 10.8 (Figure 1). To avoid any inconsistency in the illumination conditions between the photographing times, separate AGB models were established from images of each year. Non‐vegetated areas (e.g., denudated patches, pika burrows, and mounds) were mapped from the drone images using Support Vector Machine (SVM) in ArcMap at an overall accuracy higher than 94.0%, with a Kappa value over 0.89. They were used to assess the relationship between disturbance severity and AGB change.

### 2.4 Estimation of AGB

AGB was estimated from the drone images using the Red Green Blue Vegetation Index (RGBVI) ((1), (2), (3), (4)) proposed by Shi et al. (2021). After the RGBVI of the pixels (about 250) corresponding to the biomass‐harvested quarter on the ground was extracted from each RGBVI image, it was used to construct the AGB estimation model from the ground truth data.

(1) $$\mathrm{RGBVI}=\frac{\mathrm{GREEN}*\mathrm{GREEN}‐\mathrm{BLUE}*\mathrm{RED}}{\mathrm{GREEN}*\mathrm{GREEN}+\mathrm{BLUE}*\mathrm{RED}}$$

(2) $$F=\sum_{i=1}^{n}\frac{B{\;‐\;b}_{i}\;}{B\times n}$$

(3) $$\mathrm{AGB}=\;\frac{1}{t}\sum_{i=1}^{t}{\mathrm{RGBVI}}_{i}\times {\left(1‐F_{j}\right)}^{k}$$

(4) $${\mathrm{AGB}}_{B}=\mathrm{AGB}\left(\mathrm{Bare}\;\mathrm{ground}\;\mathrm{mask}\right)$$

where *n* is the number of samples, *B* is the average measured AGB of the reference plots (*G*_N_), and *b* is the measured quadrats AGB. *t* is the total number of pixels in a sampled quarter; *VI_i_* is the vegetation index value of pixel *i*; *j* denotes mowing intensity; (1 − *F_j_*)^k^ is the grazing intensity reversal factor; *k* is a constant determined via nonlinear regression (*k* = 4 produced the best result for the study area, but can be slightly different for other areas). The estimated AGB from the constructed model was modified twice, first by the mowing ratio *F* (Equations 2 and 3) which can reflect the vegetation canopy vertical properties. The second modification is to remove biomass of the bare ground area by the RGBVI of non‐vegetated pixels (Equation 4). They were determined from a newly created mask layer. The pixels were assigned a new value of zero in the output metrics and treated as the ground truth dataset. The coefficient of determination (*R* ^2^) and root mean square error (RMSE), employed to evaluate the model accuracy, were 0.88 and 19.9 g m^−2^, respectively.

### 2.5 Statistical analysis

All analyses, including model regression, ANOVA (Analysis of Variance), the least significant difference (LSD), and Pearson correlation coefficient (*r*), were implemented via Python scripting (version 3.8). ANOVA was introduced to check the potential differences of AGB between the treatments, and the least significant difference (LSD) was used to test the differences. Annual AGB changes of 2018–2019 and 2019–2020 were calculated to explore the individual and joint effects of the two disturbances. To evaluate the effects of disturbance severities on AGB change, the correlation between disturbance severities and AGB change was assessed using the Pearson correlation coefficient (*r*) (Hauke & Kossowski, 2011). *r* was also tested at each severity of both disturbances (pika population and mowing intensity). Furthermore, to quantify the exact effect of the increasing disturbance severity on annual AGB change, relative AGB change (%) between disturbance severities was calculated (Equations 5 and 6).

(5) $${\mathrm{Relative}\;\mathrm{AGB}\;\mathrm{change}\;(D}_{n,\;m})\;=\frac{\mathrm{Actual}\;\mathrm{difference}}{\left|\mathrm{Reference}\;\mathrm{value}\right|}=\;\frac{{\;D}_{m}‐D_{n}}{\left|D_{n}\right|}\times 100\%$$

(6) $${\mathrm{Relative}\;\mathrm{AGB}\;\mathrm{change}\;(D}_{m,\;h})\;=\frac{\mathrm{Actual}\;\mathrm{difference}}{\left|\mathrm{Reference}\;\mathrm{value}\right|}=\;\frac{{\;D}_{h}‐D_{m}}{\left|D_{m}\right|}\times 100\%$$

where *D* denotes AGB change caused by pika (*P*) or mowing (*M*) disturbance. In total, two disturbance gradients were assessed: none‐to‐moderate (*D*_n,m_) in Equation 5 and moderate‐to‐high (*D*_m,h_) in Equation 6. The former represents the relative AGB change of moderate disturbance severity (m) corresponding to non‐disturbance severity (n), and the latter expresses the relative AGB change of severe disturbance (h) relative to moderate disturbance (m).

## 3 RESULTS

### 3.1 Climate backdrop

**FIGURE 3:**
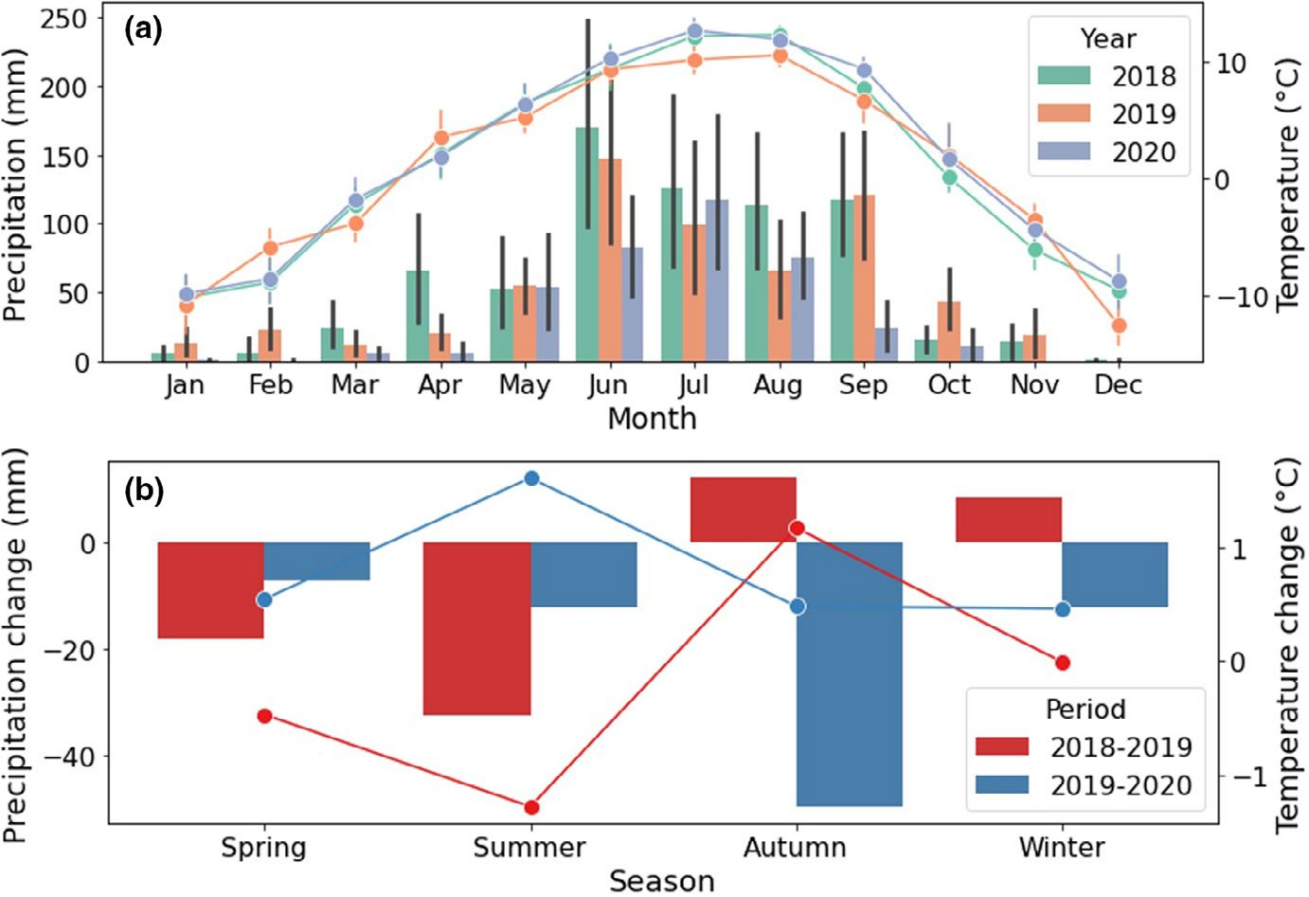
Precipitation (bars) and temperatures (lines) in experiment site (a is the monthly climate data and b is the seasonal climate data change; Spring: 1 Mar–31 May; Summer: 1 Jun‐31 Aug; Autumn: 1 Sep‐31 Nov; Winter: 1 Dec‐28 Feb)

Monthly temperature averaged from −15℃ (December 2019) to 15℃ (July 2020) over the study period (Figure 3a). Monthly mean precipitation ranged from 70 to 170 mm, concentrated in summer and early autumn (from June to Sep), and it was much lower in other months. Figure 3b summarizes the seasonal changes of temperature and precipitation over 2018–2019 and 2019–2020. In the first period, the average temperature increased by 1.1℃ in autumn but dropped in other seasons, with the most notable decrease (about −1.2℃) in summer. The seasonal precipitation declined by about 18 and 32 mm in spring and summer, respectively. Moreover, it increased slightly by about 6 and 12 mm in autumn and winter, respectively. However, seasonal temperature in the second period increased by approximately 0.4℃ in summer, with a slight decrease around 0.4℃ in other seasons. The seasonal precipitation dropped notably in this period, especially in autumn (about 50 mm), while other seasons had a drop of less than 15 mm.

### 3.2 Predicted AGB

**FIGURE 4:**
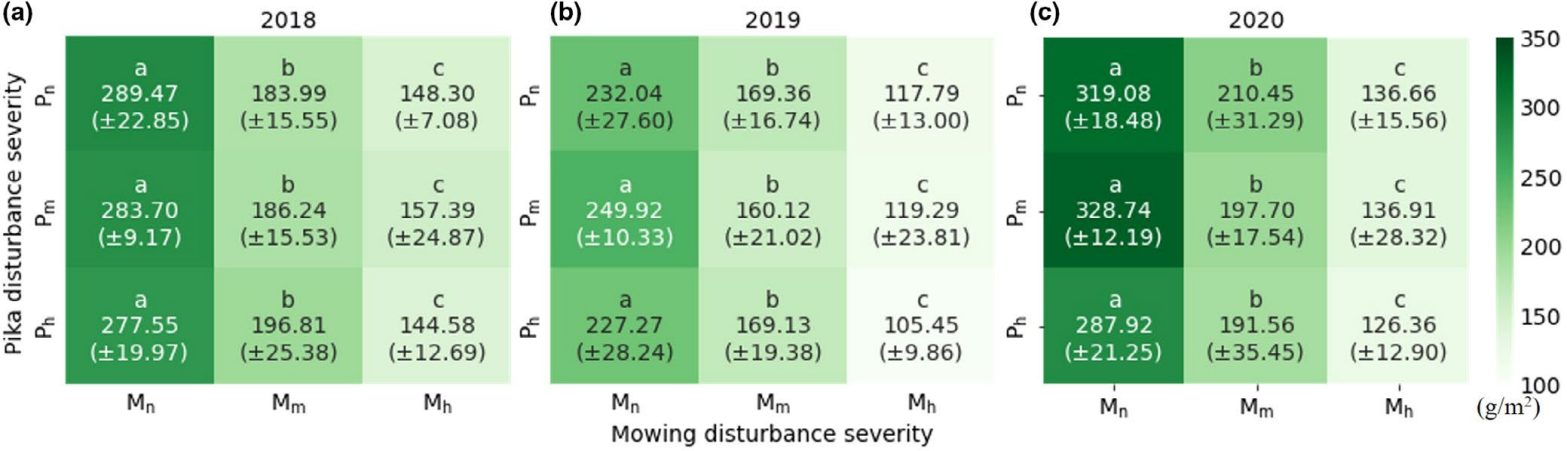
Predicted AGB for each treatment. Lowercase letters represent the significant difference (*p* < .01; numbers in bracket are standard deviation; a, b, c are the predicted AGB for 2018, 2019 and 2020, respectively)

Figure 4 shows that all treatments always had a consistent rank of AGB and a similar level of significant difference throughout the three years (LSD, *p* < .01). In 2018, the reference plot (*P*_n_ *M*_n_) had the highest AGB of 289.47 g m^−2^ (equivalent to 217.11 kg dry matter per plot). The other two non‐mowed plots (*P*_m_ *M*_n_ and *P*_h_ *M*_n_) had an equivalent AGB of around 280 g m^‐−2^, while the moderately mowed plots (*M*_m_) achieved a much lower AGB of about 190 g m^−2^, and the heavily mowed plots (*M*_h_) yielded the lowest AGB of around 150 g m^−2^. Moreover, AGB was not significantly different between pika disturbance severities at the same mowing intensity, but it varied significantly between the three mowing intensities at every pika disturbance level. This finding still holds true in the other 2 years. The AGB rank and the significant difference were the same regardless of the absolute quantity. In 2019, *P*_m_ *M*_n_ had the highest AGB of 249.92 g m^−2^, followed by *P*_n_ *G*_n_ of 232.04 g m^−2^ (equivalent to 174.03 kg dry matter per plot) and *P*_h_ *M*_n_ of 227.27 g m^−2^. Both *M*_m_ and *M*_h_ plots had a lower AGB of 160–170 g m^−2^ and 105–120 g m^−2^, respectively. In 2020, AGB increased markedly, but the significance among different treatments remained unchanged. Namely, the *M*_n_ plots had the highest AGB from 287.92 to 328.74 g m^−2^, and the *M*_h_ plots had the lowest AGB from 126.36 to 136.91 g m^−2^. Among them, the reference plot had the highest AGB of 319.08 g m^−2^, equivalent to 239.31 kg dry matter per plot.

### 3.3 AGB change

#### 3.3.1 Annual AGB change under sole disturbance

**FIGURE 5:**
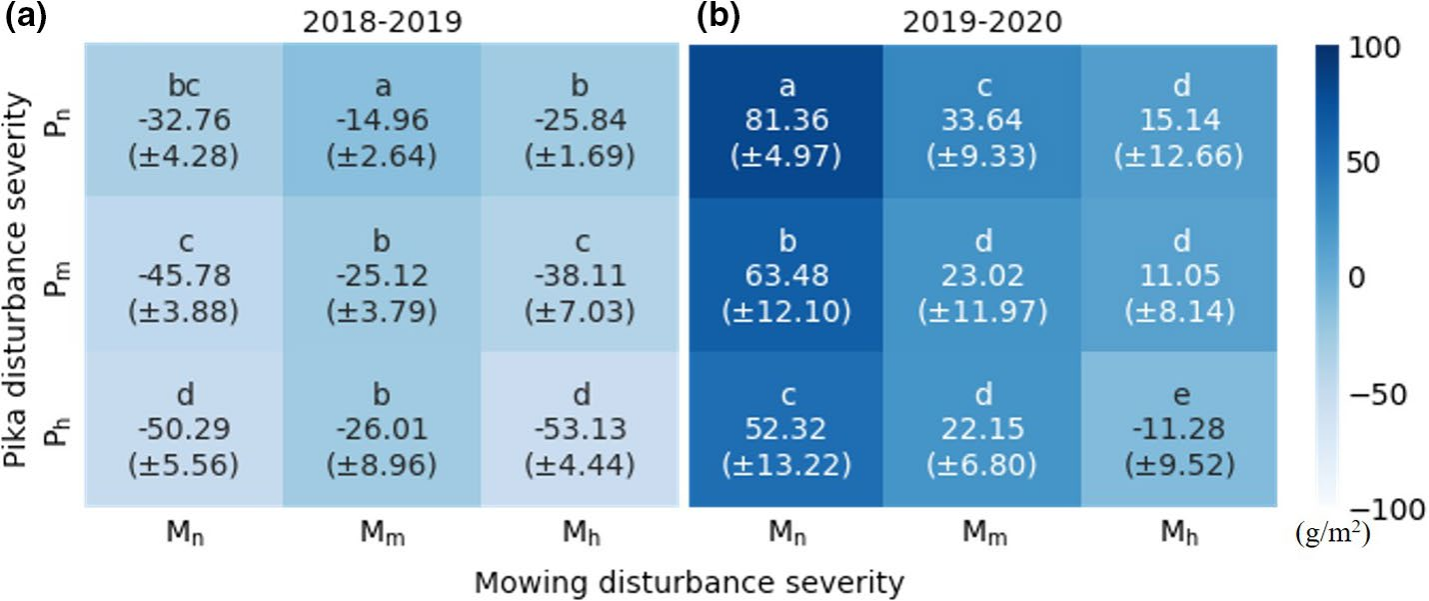
Heat map of AGB change for the different disturbance severity treatments. Lowercase letters represent the significant difference (*p* < .01; numbers in bracket are standard deviation; a, and b are the AGB change for 2018‐2019 and 2019‐2020, respectively)

Figure 5 shows AGB changes in relation to disturbance severities in 2018–2019 and 2019–2020, with their significance level indicated by lower case letters (LSD, *p* < .01). Over the two periods, the first column from the left and the first row from the top show the sole disturbance of pika and mowing, respectively. The reference plot (*P*_n_ *M*_n_) was counted in the plots solely disturbed by pika or mowing.

Generally, all treatments had negative annual AGB changes during 2018–2019, but positive changes during 2019–2020 except *P*_h_ *M*_h_. In the first period, non‐pika plots (*P*_n_), which suffered only the sole effect of mowing, experienced a fluctuating trend in AGB change from −32.76 g m^−2^ rising to −14.96 g m^−2^ and then declining to −25.84 g m^−2^ as the mowing severity rose from low to medium and then to high. However, mowing itself caused AGB change to decrease from 81.34 g m^−2^ to 33.64 and 15.14 g m^−2^ for moderate and severe mowed plots, respectively, in the second period. The first column from the left (*M*_n_), which showed the sole effect of pika disturbance, had a noticeable drop in AGB change from −32.76 to −50.29 g m^−2^ as the pika disturbance severity rose from low to high in the first period. Although AGB increased in the second period, it had a downward trend from 81.34 to 52.32 g m^−2^ along with the rising pika disturbance severity. These results indicate that more severe pika disturbances decreased annual AGB change in both periods, but mowing decreased AGB change only in the second period. The mowed plots had less decrease than the reference plots in the first period.

#### 3.3.2 Annual AGB change under joint disturbances

Annual AGB changes under joint disturbances are shown in Figure 5, including *P*_m_ *M*_m_, *P*_m_ *M*_h_, *P*_h_ *M*_m_, and *P*_h_ *M*_h_. During 2018–2019, the most disturbed plots of *P*_h_ *M*_h_ had the largest decrease of −53.13 g m^−2^ in AGB change, which was significantly lower than −38.11 g m^−2^ of *P*_m_ *M*_h_. In the *M*_m_ column, both *P*_m_ *M*_m_ and *P*_h_ *M*_m_ treatments had a similar AGB change of around −26 g m^−2^. Although the jointly disturbed plots were subject to both disturbances, their AGB change decreased less than that of the plots solely disturbed by pika (*M*_n_). During 2019–2020, the *M*_h_ column had the smallest value of AGB change, among which the severely disturbed plots (*P*_h_ *M*_h_) had a decrease of 11.28 g m^−2^, against an increase of 11.05 g m^−2^ in the *P*_m_ *M*_h_ plots, while AGB in the *P*_m_ *M*_m_ and *P*_h_ *M*_m_ plots was about 23 g m^−2^ higher. Moreover, joint disturbances affected AGB change more negatively than sole disturbances in the second period, as revealed by their values and significances. Overall, a more severe disturbance level can amplify the effect of a disturbance on AGB decrease, on the order of *P*_m_ *M*_m_ < *P*_h_ *M*_m_ < *P*_m_ *M*_h_ < *P*_h_ *M*_h_. Among them, *P*_h_ *M*_h_ had a significant difference from the others. *P*_m_ *M*_h_ had more negative effects than *P*_h_ *M*_m_, indicating that severe mowing decreased AGB change more than severe pika disturbance.

#### 3.3.3 Relative annual AGB change

The exact effect of rising mowing disturbance severity on annual AGB change at each pika disturbance severity was evaluated in terms of the relative AGB change in the two gradients of *M*_n,m_ and *M*_m,h_ (Table 1). During 2018–2019, the relative annual AGB change was around 50% in *M*_n,m_ (the actual difference is about 20 g m^−2^). It became negative in *M*_m,h_, with a value of −72.2%, −51.7%, and −104.2% for *P*_n_, *P*_m_, and *P*_h_, respectively. Correspondingly, AGB change decreased by 10.87 g m^−2^ for *P*_n_, 12.99 g m^−2^ for *P*_m_, and 27.56 g m^−2^ for *P*_h_ in this gradient. However, all relative AGB changes were negative during 2019–2020. In *M*_n,m_ gradient, the values were −58.5% (−44.27 g m^−2^), −57.2% (−41.57 g m^−2^), and −53.6% (−31.56 g m^−2^) at the three pika severities, respectively. For the *M*_m,h_ gradient, relative AGB change was around −50% for *P*_n_ and *P*_m_, with varied actual differences of −21.56 g m^−2^ and −9.96 g m^−2^, respectively. However, *P*_h_ had the smallest relative AGB change of −149.5% (−31.19 g m^−2^). Relative AGB changes among the three pika severities were less different in *M*_n,m_, but they were more varied in *M*_m,h_ for both periods. *P*_h_ in *M*_m,h_ had a double and triple negative effect on biomass change relative to *P*_n_ and *P*_m_ in the first and second period, respectively. These results indicate that a higher pika disturbance level increased biomass loss in the M_m,h_ gradient. This effect was amplified in the second period.

**TABLE 1:** Relative AGB change (%) between mowing disturbance severities

Pika severity	2018–2019		2019–2020	
	*M*_n,m_	*M*_m,h_	*M*_n,m_	*M*_m,h_
*P*_n_	54.3 (17.80)	−72.2 (−10.87)	−58.5 (−44.27)	−54.0 (−21.56)
*P*_m_	45.1 (20.66)	−51.7 (−12.99)	−57.2 (−41.57)	−47.8 (−9.96)
*P*_h_	48.3 (24.27)	−104.2 (−27.12)	−53.6 (−31.56)	−149.5 (−31.19)

Note

Numbers in the bracket represent actual differences of AGB change (g m^−2^).

In comparison, the intensification of pika disturbance always decreased annual AGB change regardless of mowing severity in both periods (Table 2). The rising pika disturbance severity from none to medium (*P*_n,m_) decreased annual AGB change with percentages ranging from −67.9% to −39.7% in 2018–2019, and from −37.0% to −25.1% in 2019–2020. Generally, relative AGB changes in *P*_m,h_ gradient were less than those in *P*_n,m_. Specifically, in the first period, relative AGB change was only −9.9% and −3.6% for *M*_n_ and *M*_m_, respectively; but it was amplified about fourfold in *M*_h_ (−39.4%). The changes were similar in the second period except the amplification was about 20‐fold, which was much higher than that in the first period. This relativity remains unchanged in terms of the actual differences in AGB change. These results indicate that a higher mowing intensity boosted biomass loss in the *P*_m,h_ gradient. This effect was magnified in the second period. Additionally, this loss was notably larger than the biomass loss induced by severe pika disturbance in the *M*_m,h_ gradient.

**TABLE 2:** Relative AGB change (%) between pika disturbance severities

Mowing severity	2018–2019		2019–2020	
	*P*_n,m_	*P*_n,m_	*P*_n,m_	*P*_n,m_
*M*_n_	−39.7 (−13.02)	−9.9 (−4.51)	−30.3 (−17.88)	−9.7 (−5.17)
*M*_m_	−67.9 (−10.16)	−3.6 (−0.89)	−25.1 (−10.84)	−2.1 (−0.49)
*M*_h_	−47.5 (−12.27)	−39.4 (−15.02)	−37.0 (−13.58)	−192.8 (−23.05)

Note

Numbers in the bracket represent actual differences indifference AGB change (g m^−2^).

### 3.4 Correlation of AGB change with disturbances

The quantitative relationship between disturbance severity (pika population and mowing intensity) and AGB changes is presented in Table 3. Pika population had a poor relationship with AGB changes in all treatments, with a Pearson coefficient (*r*) of −.471 and −.368 for two periods, respectively. However, at a given mowing severity, pika population had a close negative relationship with AGB change, achieving a coefficient from −.779 to −.626 in the first period, and from −.884 to −.733 in the second period. Similar results were also obtained if judged by *r* between bare area and AGB, and between bare area change and AGB change (Table 4). Such a relationship is due to the strong relationship (*r* > .7) between pika population and bare area change (Table 5).

**TABLE 3:** Pearson correlation coefficient (*r*) between disturbance severity and AGB change

Correlations	Treatments		Period	
			2018–2019	2019–2020
Pika population vs AGB change	Mowing severity	None	−.779	−0.733
		Medium	−.769	−0.884
		High	−.626	−.818
	All		−.471	−.368
Mowing intensity vs AGB change	Pika severity	None	.328	−0.927
		Medium	.295	−0.911
		High	.090	−0.939
	All		0.120	−.849

**TABLE 4:** Pearson correlation coefficient (*r*) between bare area and AGB at each mowing severity

Correlation	Period/Year	Mowing Severity			All
		None	Medium	High	
Bare area vs AGB	2018	−.853	−.855	−.893	.274
	2019	−.871	−.894	−.958	.011
	2020	−.880	−.929	−.944	−.040
Bare area change vs AGB changes	2018–2019	−.663	−.808	−.719	−.233
	2019–2020	−.822	−.879	−.836	−.323

**TABLE 5:** Pearson correlation coefficient (*r*) between disturbance severity and bare area changes

Input data	Pearson correlation coefficient (*r*)	
	2018–2019	2019–2020
Pika population	.809	.719
Mowing intensity	.232	.033

Mowing intensity had different correlations with AGB change in the two periods (Table 3). It was hardly correlated with AGB change in all treatments in the first period (*r* = .12), but highly correlated in the second period (*r* = −.849). Such different impacts in two periods can be explained by the abnormal AGB drop in the reference plot in the first period. However, the relationship between mowing intensity and AGB change was less influenced by pika disturbance severity, given that *r* at each pika population was equivalent to that of all treatments (.090 < *r* < .328 in the first period, −.939 < *r* < −.911 in the second period). Overall, mowing intensity weakened the relationship between pika population and AGB change, but pika disturbance hardly affected the relationship between mowing severity and AGB change.

## 4 DISCUSSION

### 4.1 Climate effect

Climate conditions are key controls upon the variability of grassland AGB on the QTP (Liu & Chen, 2000; Zhong et al., 2019). AGB of the reference plot without any disturbance was mainly influenced by climate. Generally, the reference plot had the lowest AGB (232.04 g m^−2^) in 2019 but the highest in 2020 (319.08 g m^−2^) as shown in Figure 4. Over the 3 years, 2019 had less monthly precipitation and a lower month temperature in spring and summer than 2018, which are the main limiters of grass growth, resulting in a lower AGB in 2019 (Wingler & Hennessy, 2016). On the other hand, although 2020 had a slightly reduced precipitation in summer and spring, this year had a much warmer temperature than 2019, leading to a higher accumulative AGB, because a warmer temperature, especially in the growing season, can stimulate grass growth (Hu et al., 2016; Na et al., 2011). Furthermore, as the study area is located on a floodplain, the availability of groundwater weakens the importance of precipitation upon vegetation growth. Moreover, seasonal mean precipitation increased in autumn and winter in 2019, plants' demand for water decelerated in these two seasons caused by the cold temperature stressing on plant growth (Sun et al., 2015; Zhang, Kong, et al., 2018), but the storage of rainfall in the soil can promote plant growth in the next year (Guo et al., 2015; Mueller et al., 2016).

### 4.2 Comparison of disturbance effects

#### 4.2.1 Mowing effects

In this study, mowing directly affected the absolute quantity of AGB, and indirectly influenced its pace of change over time. Mowing can promote grass biomass by stimulating plant growth. Accordingly, the mowed meadow can produce more accumulated biomass than the un‐mowed meadow, but only slightly (Bernhardt‐Römermann et al., 2011; Ten Cate & Gusewell, 2003). Due to the regulated clipping height, the effect of mowing on plant height is universal throughout all the plots. As the mowed plots have a lower AGB with less tissue for photosynthesis, they are more subject to the effect of climate stresses than un‐mowed plots, and it takes a longer time for the remaining biomass to reach a higher level (Bernhardt‐Römermann et al., 2011). Furthermore, if the grass cover was spatially uniform, then the clipped biomass from a plot would also be spatially uniform, but less if the grassland contained more bare patches (Pang & Guo, 2017; Qin et al., 2020). This is demonstrated by the close negative linear relationship between bare area change and AGB change at every mowing severity (Table 4).

Generally, disturbed grassland has less biomass than undisturbed grassland (Fu et al., 2012; Qin et al., 2014; Zhang et al., 2016). However, mowing had an opposite effect on annual AGB change in the two periods (Figure 5). This result can be explained by the decrease of AGB in the reference plot which had low AGB and short vegetation height in 2019. All the mowed plots had the same mowing intensity over 3 years, but the amount of clipped grass varied from year to year because different plants had their own natural heights in response to climate conditions (Pöyry et al., 2006). Vertically, the allocation of grassland biomass has a pyramid‐like distribution, with a higher biomass density closer to the ground (Tackenberg, 2007), 40%–60% of the total biomass was distributed in 0–10 cm above the ground, about 30% in 20–30 cm, and less than 20% over the 30 cm height in a meadow in Ireland (Ten Cate & Gusewell, 2003). Thus, the mowed plots contained more biomass than un‐mowed plots, resulting in less annual AGB change. In comparison, the un‐mowed plots had different vegetation heights every year, and taller plants had more biomass, leading to a negative annual AGB change in the climate‐stressed period. Obviously, mowing was unable to increase annual AGB change. On the contrary, AGB change of the mowed plots was sensitive to climate variability which is the key control upon alpine grassland productivity (Benot et al., 2014; Liu & Chen, 2000; Piseddu et al., 2021; Yang et al., 2012).

Inevitably, mowing‐simulated grazing differs from livestock grazing in that livestock preference for palatable species results in an uneven height of grass (Isselstein et al., 2007). In contrast, mowing leads to a uniform height. Besides, AGB grazed by livestock is mostly recycled back into the ecosystem through excretion (Long et al., 2008). Also, livestock trampling can influence grass growth and expand bare area by enhancing soil bulk density, also decreasing hydraulic conductivity and respiration rate (Chai et al., 2019). Generally, livestock grazing has more positive influences on grassland biomass than mowing, but the differences between the two are rather small (Talle et al., 2016). A comparison between livestock grazing and mowing in a mountain grassland reveals that they have little differences in changing plant species diversity, but livestock grazing has twofold higher AGB loss than mowing under drought tress (Deléglise et al., 2015). Additionally, mowing based on clipping height is different from livestock grazing which is based on a stable intake amount of forage (Xin‐wei et al., 2005). It is recommended that artificial grazing simulated by mowing should use the removed grass height or biomass as the indicator for artificial grazing intensity.

#### 4.2.2 Pika effects

Due to pika's complex feeding habit such as gnawing roots, seeds, and caching forage (Kang et al., 2019), pika‐consumed biomass cannot be factored in the AGB estimation. However, biomass loss associated with bare area enlargement caused by pika can be accurately estimated (Shi et al., 2021), owing to the strong relationship of bare area change with AGB change (Table 4). Generally, an adult pika devours 77.3 g of fresh grass/day (Fan et al., 1999), equivalent to 11.37 kg dry matter per annum. In this study, the biomass loss caused by pika (*M*_n_ of *P*_n,m_ gradient for example) was at 13.02–17.88 g m^−2^ (equivalent to 1.40–1.92 kg per pika annually). This figure is much lower than the directly consumed biomass, but the loss is non‐recoverable as pika likely destroyed the grass root system via its burrowing activities (Yu et al., 2017; Zhang et al., 2003). Furthermore, the fenced plots may not encompass sufficient palatable species of grasses, forcing pika to seek more food via digging and gnawing (Guo et al., 2012; Hogan, 2010). However, the doubling of pika population from 7 to 14 individuals per plot did not double the annual AGB change because multiple pikas can share food and burrow (Dobson et al., 1998; Fan et al., 1999). On the other hand, pika population still had a positive relationship with bare area change (Table 5), but the increase in the bare area slows down at a higher pika population (Sun et al., 2016). A good correlation existed between pika population (bare area) and AGB change at each mowing severity (Tables 3 and 4). However, these relationships disappeared when all the plots were included in the regression. This disappearance can be explained by the varied AGB change between the three mowing severities according to significant difference tests. Similar work in an alpine meadow on the QTP reported by Li et al. (2019) did not find any relationship between pika population and AGB that was decreased mainly by livestock, but their work did not explore the relationship to each grazing intensity. Thus, the effect of pika disturbance on alpine grassland biomass was subject to mowing severity that overwhelmed the relationship between pika disturbance and AGB change. Conversely, some authors note that pika is a keystone rodent for increasing grassland biomass (Liu et al., 2017; Smith & Foggin, 1999), with herbs occupying more than 90% of bare patches (Li et al., 2014; Wei et al., 2007). Despite this increase, however, most of the newly grown biomass was inedible for livestock, and some even poisonous (Wen et al., 2010). Further study is required to investigate the change of plant species in different pika disturbance severities.

#### 4.2.3 Joint effects

A more severe disturbance level can amplify the effect of another disturbance on AGB decrease, and the joint effects of both mowing and pika disturbances can amplify the negative effect of either disturbance on AGB change. In particular, a high pika disturbance level boosted biomass loss as the mowing intensity rose from moderate to high. In greater numbers, pikas face more competition for food and living space, which drives them to create more bare patches (e.g., gnawing at grassroots), leading to more biomass loss (Zhang et al., 2016). Also, the impact of pika on landscape fragmentation was higher than mowing in this experiment in another study (Li et al., 2021). These effects were amplified in the second period because bare area expanded rapidly once it was initiated (Li, Perry, et al., 2013). Meanwhile, all low pika disturbances, not exceeding the moderate level, had a similar influence on AGB change even if mowing intensity increased to the high level. Therefore, the density of 14 pikas per plot (about 200 pikas per hectare) surpassed the threshold of pika outbreak in the severely mowed plots, but not in the plots of low mowing intensity (none and moderate mowed plots). Indeed, pika has been reported to have a positive effect on biomass in the grazing‐adapted alpine grassland (Qin et al., 2020), but excessive pika disturbance may devastate grassland AGB and health, especially in overgrazed grasslands (Sun et al., 2009).

Generally, mowing indirectly influences pika by reducing grass height and changing vegetation structure (Bernhardt‐Römermann et al., 2011). However, intensive mowing beyond the moderate level boosted biomass loss, and this loss would be magnified over time in response to continuous disturbances. This can be explained by the change in vegetation height. Severely mowed plots lost most tissues and the remaining grasses have difficulties in photosynthesis, especially in a stressed climate (Chen et al., 2014; Piao et al., 2011; Xu et al., 2013). On the other hand, plant height can impact pika burrowing activities in that lower plants can lead to a higher burrow density and more bare patches (Wang et al., 2010), because pikas will dig more holes to hide and escape from predators rapidly (Fan & Zhang, 1996; Wangdwei et al., 2013). Furthermore, pikas will cut down plants to create an open view for safety checking and danger warning (Smith & Foggin, 1999). Additionally, mowing intensity has significant impacts on grassland AGB under different climate conditions. In this study, the relative importance of rainfall was the highest at high mowing intensity and the contribution of temperature was greatest at low mowing intensity (Bernhardt‐Römermann et al., 2011). However, research by Liu et al. (2018) reported that mowing was able to decrease the damaged area by 14% in a grassland in Inner Mongolia, but differences between varied mowing intensities were not considered in this work. In this study, the obtained results demonstrate that severe mowing is conducive to rodent outbreaks. Severe mowing led to more loss of AGB than severe pika disturbance in heavily disturbed plots. Severe pika disturbance caused no obvious differences in AGB change between moderately mowed and intact plots, but had much higher negative effects on the AGB change in severely mowed plots. Therefore, reducing mowing severity is the primary choice to improve AGB in the plots heavily disturbed by mowing and pika, and pikas do not pose a threat if the grassland is mowed at a low intensity.

In reality, livestock and pika have coexisted for thousands of years in grazing‐adapted alpine grassland (Badingquiying et al., 2018). However, both grazing and rodent disturbances had negative impacts on grassland biomass (Chen et al., 2007; Pang & Guo, 2018), and grazing has been reported as an important factor in causing pika outbreaks (Foster et al., 2014; Su et al., 2016). Thus, based on findings from this study, it is more effective to reduce grazing intensity than control the pika population to restore the severely disturbed grassland. It will be interesting to see if grazing combined with pika disturbance will have the same effects on AGB change in a natural alpine grassland as those found in this controlled experimental study. Inevitably, alpine grassland AGB change is subjective to the complex interplay and associated uncertainties of multiple stressors; besides, livestock and pika disturbances, including interactions between climate and land‐use change (Carlyle et al., 2014; Hovenden et al., 2014), environmental legacy effects (De Boeck et al., 2018; Gong et al., 2020), and stages of degradation or recovery of grasslands (Guo et al., 2019) also play important roles in AGB and its change.

## 5 CONCLUSIONS

This controlled study quantified the singular and joint effects of mowing and pika disturbances on alpine grassland AGB changes on the QTP for the first time. Pika‐induced AGB change through bare area expansion was much smaller than their daily consumption, but their damage on grassland was hardly recoverable, and this effect can be amplified with time. Moreover, pika's effects on AGB change were always overwhelmed by mowing severities, but it can be identified by inspecting it into each mowing intensity. Mowing intensity weakened the relationship between pika population and AGB change, but pika disturbance hardly affected the relationship between mowing severity and AGB change. The joint effect of both disturbances amplified the negative effect of individual disturbances on AGB. The damage of high mowing severity on AGB loss was larger than high pika severity in the heavily disturbed grassland. Pika disturbances induced less difference in AGB change if mowing did not exceed the moderate level. Thus, reducing mowing intensity is more effective than controlling pika population for boosting AGB production in heavily disturbed plots. Based on the findings of this study, we recommend that reducing livestock grazing intensity rather than controlling pika population should be the primary method to improve AGB in the real livestock grazing grasslands on the QTP.

## CONFLICT OF INTEREST

The authors declare that they have no known competing financial interests or personal relationships that could have appeared to influence the work reported in this paper.

## AUTHOR CONTRIBUTIONS

**Yan Shi:** Data curation (equal); Formal analysis (lead); Methodology (lead); Writing – original draft (lead). **Jay Gao:** Formal analysis (supporting); Methodology (supporting); Supervision (lead); Writing – original draft (lead); Writing – review & editing (lead). **Xilai Li:** Conceptualization (lead); Data curation (equal); Funding acquisition (lead); Methodology (supporting); Writing – review & editing (supporting). **Jiexia Li:** Data curation (equal); Writing – review & editing (supporting). **Gary Brierley:** Writing – review & editing (equal).

## Data Availability

The biomass and climate data are available in Dryad: https://doi.org/10.5061/dryad.p5hqbzkr2.
